# CRISPR in *Nucleic Acids Research*: the sequel

**DOI:** 10.1093/nar/gkae159

**Published:** 2024-03-27

**Authors:** Julian E Sale, Barry L Stoddard

**Affiliations:** MRC Laboratory of Molecular Biology, Francis Crick Avenue, Cambridge CB2 0QH, UK; Division of Basic Sciences, Fred Hutchinson Cancer Center, 1100 Fairview Ave. N., Seattle, WA 98109, USA

In 2016, *Nucleic Acids Research* (‘NAR’) assembled a special collection of studies, published in its pages over the preceding five-years, that described the biology and use of CRISPR/Cas systems (1). The 79 manuscripts presented in that collection included 37 studies that described the evolution, genetics, and mechanisms of CRISPR defense systems and an almost equal number of studies describing their development and application for gene modification and genome engineering. Those publications were supplemented with several descriptions of related online computational tools and two review articles that summarized the rapidly growing body of literature surrounding the evolution of CRISPR systems (2) and their application for gene editing and genome engineering (3).

That collection of publications appeared in our pages over a four year period that followed the first publications (one at NAR and several others elsewhere) that initially documented the composition, mechanism of action, and potential for targeted gene editing by CRISPR-Cas9 (4–7). Those papers remain organized and available online at https://academic.oup.com/nar/pages/crispr_cas_collection; together they provide a snapshot into the early days of the ‘CRISPR Craze’ (8) via the content in one journal. That collection was led by one of the first demonstrations of the biological role of CRISPR as a microbial antiviral defense system and corresponding details of its biochemical mechanism (4), followed by subsequent manuscripts describing early studies of how gene-targeting spacer elements are acquired and embedded within CRISPR loci (9,10), equally early descriptions of auxiliary protein and RNA components involved in the assembly and organization of CRISPR systems (11,12), the biophysical and structural basis of a CRISPR system (13), several demonstrations of CRISPR being applied for gene editing and genome engineering in various model organisms (14–19), and early descriptions of using CRISPR for targeted gene activation and repression (14) or epigenetic modification (20).

The editorial published alongside that special collection (1) concluded by stating that ‘Moving forward, the field of CRISPR biology and applications clearly remains fertile ground for continued study. Many details of the mechanisms employed by CRISPR systems…and particularly those of the more complex multi-protein systems, remain to be elucidated. Variants of the prototypical Cas9 nuclease, including those with substantially different domain sizes, unique patterns of structural organization, and distinct reaction mechanisms yielding differing DNA product ends, offer the possibility of unique properties and uses for biotechnology. *Nucleic Acids Research* looks forward to continuing to receive and publish such studies, and to the expansion of our online collection of CRISPR studies as the field continues to grow and mature.’

Now, eight years later and as part of NAR’s 50th anniversary celebration, we have reviewed our subsequent content and identified almost 500 articles focused upon or utilizing CRISPR systems that have been published in NAR since that early collection. As in 2016, these more recent studies describe both the native biological functions and mechanisms displayed by CRISPR systems in their natural hosts, and the greatly expanded use of CRISPR technology for basic research, synthetic biology and potential therapeutic applications. The titles that we have chosen to highlight (https://academic.oup.com/nar/pages/crispr-collection-2024) represent some of the most impactful of these studies. Together, they provide a fresh view of how the study and use of CRISPR has grown since its earliest frenetic days of discovery and innovation, and how it has expanded the possibilities for biological research to an extent that would have stunned even the most forward-thinking investigator at the time when the composition and possible purpose of CRISPR loci were first described in detail (21–23).

Whereas all of the early CRISPR papers found in the pages of NAR up through mid-2016 could be presented in a single collection, and easily divided into two broad areas of study, the studies published in NAR since then, that have either been focused on CRISPR or have made significant use of it, are far too numerous to present in their entirety, and far too diverse in their scope to categorize simply as either ‘biological studies’ or ‘applications’. Therefore, we have selected a subset of 72 publications published from 2016 onwards that have garnered exceptional numbers of views, downloads, and citations, and/or that reflect the growing range of research and development enabled by CRISPR technology.

Recently published studies of native CRISPR biology span evolution, mechanisms, structures, biophysical behavior, and the ultimate role of CRISPR systems in cellular and genomic defense during the unending confrontations between microbes and the viruses that target them. These papers include *in vitro* analyses (spanning CryoEM and crystallographic studies, single particle biophysical analyses, and a wide range of enzymatic and biochemical examination of their form and function) and a similar number of *in cellulo* studies further examining function and behavior—or in many cases *in vitro* and *in cellulo* studies of CRISPR function within a single manuscript. Those publications are joined by variety of bioinformatic analyses of CRISPR system distribution and diversity, as well as expanded online tools for their study and application.

One of the most recent and rapidly growing areas of investigation into CRISPR biology focuses upon the ways in which phage combat and evade their action and defensive capabilities. Our first published study of an phage-encoded resistance factor (i.e. an ‘anti-CRISPR’) appeared in 2018 (24); since then, many more additional studies of anti-CRISPRs have been described in our pages. That first study and one additional publication have been selected for inclusion in this special collection (24,25).

Over the same time span, nearly seventy publications have been published in NAR describing the use of CRISPR systems for gene editing and genome engineering. Since 2016, the manner in which CRISPR has been applied for such purposes has diversified enormously, as reflected by recent publications in the journal describing the use of CRISPR for targeted DNA methylation (20,26) or demethylation (27), editing and correction of disease-associated genetic loci (28,29), gene editing and genome engineering in prokaryotes (30–32) or in single cell eukaryotes (33,34), as well as the refinement of prime editing systems (35) and the use of CRISPR for large-scale genome engineering and assembly (36). Those demonstrations have been accompanied by an equally broad range of new methods for the use of CRISPR in biotechnology. Those reports have described novel strategies for the improvement of guide RNA scaffolds (37), the temporal and spatial control of CRISPR activity (38,39), the packaging and delivery of CRISPR to defined cellular and/or *in vivo* targets (40–42), the control of DNA editing and repair outcomes induced by CRISPR action (43,44), the analysis and improvement CRISPR specificity, activity, and on-target versus off-target action (45,46), RNA editing (47), and the development of platforms for genetic imaging, detection and diagnostics (48–50).

While many of the studies published in NAR provide new details regarding CRISPR function and application, it is perhaps the increasingly routine use of CRISPR for everyday studies throughout molecular and cellular biology that most clearly reflects the extraordinary impact these molecular tools have had on the biological research community. Of the papers published at NAR over the past eight years that clearly involve CRISPR in some fashion, a significant number have employed it for basic studies ranging from the examination of *cis-* and *trans-*acting factors that regulate and control gene expression, detailed studies of DNA damage response and repair pathways, or the dissection of genetic and molecular pathways involved in physiology, metabolism, development and disease. In particular, we note the sudden appearance and now quite common use of high-throughput CRISPR screens for many purposes. A significant number of such studies have facilitated early-stage pharmacological studies of molecular drug targets and corresponding cellular phenotypes that might have previously required years of effort and resources only found within industry. Three of the first such publications in NAR (51–53) have been selected for the collection from a much larger group of studies, all of which have been published since 2018.

Moving forward, the editors look forward to seeing what the next few years of biological research will yield as a result of CRISPR, in combination of other transformative technologies. In addition to what CRISPR has delivered, seemingly almost overnight, to investigators’ doorsteps, researchers are now exploiting similar revolutions in their ability to sequence nucleic acids, perform single-cell analyses, determine the structures of complex biomolecular machines, design and fabricate entirely novel molecules with defined biological functions, and leverage extraordinary information technology capabilities. Considered together, these revolutions are creating a new era of easily accessible, democratized research programs and corresponding potential for discovery that is genuinely astonishing. We anticipate that future editors, authors and readers of *Nucleic Acids Research*, hopefully in another 50 years, will look back on this era and summaries such as this with a combination of amazement, appreciation, and maybe even some amusement as they consider and describe their own past, present, and future as research investigators.
